# Usability and Acceptance of the Librarian Infobutton Tailoring Environment: An Open Access Online Knowledge Capture, Management, and Configuration Tool for OpenInfobutton

**DOI:** 10.2196/jmir.4281

**Published:** 2015-11-30

**Authors:** Xia Jing, James J Cimino, Guilherme Del Fiol

**Affiliations:** ^1^ Department of Social and Public Health Ohio University Athens, OH United States; ^2^ Informatics Institute University of Alabama at Birmingham Birmingham, AL United States; ^3^ Department of Biomedical Informatics University of Utah Salt Lake City, UT United States

**Keywords:** clinical decision support systems/instrumentation, evaluation studies as topic, knowledge management tool, The Librarian Infobutton Tailoring Environment

## Abstract

**Background:**

The Librarian Infobutton Tailoring Environment (LITE) is a Web-based knowledge capture, management, and configuration tool with which users can build profiles used by OpenInfobutton, an open source infobutton manager, to provide electronic health record users with context-relevant links to online knowledge resources.

**Objective:**

We conducted a multipart evaluation study to explore users’ attitudes and acceptance of LITE and to guide future development.

**Methods:**

The evaluation consisted of an initial online survey to all LITE users, followed by an observational study of a subset of users in which evaluators’ sessions were recorded while they conducted assigned tasks. The observational study was followed by administration of a modified System Usability Scale (SUS) survey.

**Results:**

Fourteen users responded to the survey and indicated good acceptance of LITE with feedback that was mostly positive. Six users participated in the observational study, demonstrating average task completion time of less than 6 minutes and an average SUS score of 72, which is considered good compared with other SUS scores.

**Conclusions:**

LITE can be used to fulfill its designated tasks quickly and successfully. Evaluators proposed suggestions for improvements in LITE functionality and user interface.

## Introduction

### Infobutton, OpenInfobutton, Meaningful Use and Librarian Infobutton Tailoring Environment

Studies show that clinicians raised about 1 question for every 2 patients seen and that over 60% of these questions are not answered [1-4]. Meeting clinicians’ information needs in a timely manner at the point of care helps them make more informed decisions and provide better quality care to patients [1]. An infobutton is a clinical decision support tool embedded within electronic health record (EHR) systems that has been shown to be an effective tool to meet such information needs [5,6].

Infobuttons are enabled within EHR systems through a Web service known as *infobutton manager.* An infobutton manager is an application accessible from within EHR systems that provides EHR users with context-specific links (CSLs) to external knowledge resources when an EHR user clicks on an infobutton in a particular clinical context. An infobutton manager uses a knowledge base to manage all CSLs. An example of an infobutton manager is available in OpenInfobutton [7], which consists of a suite of open source tools to help various health care organizations and EHR developers to implement HL7-compliant infobuttons.

Meaningful Use [8] is a US government incentive program to encourage health care organizations to adopt certified EHR technology and demonstrate its usage in meaningful ways. Two of the core measures of Meaningful Use regulations for eligible professionals and hospitals are to deliver patient-specific education materials and to utilize clinical decision support interventions to improve performance. Both measures can be achieved using infobutton functionality encompassed in certified EHR systems, which are compliant with the *HL7 Context-Aware Knowledge Retrieval (infobutton) Standard* [9,10]. There are over 1000 certified EHR products with HL7-compliant infobutton functionality. However, how to populate, manage, and customize infobutton managers’ knowledge bases easily and in bigger scale to optimize infobutton’s performance after implementation have not yet been solved.

Librarian Infobutton Tailoring Environment (LITE) [11], a part of OpenInfobutton, is the tool that enables knowledge resource experts (eg, medical librarians) to populate, manage, and customize infobuttons’ knowledge bases via graphical user interfaces according to different health care organizations’ characteristics.

OpenInfobutton is maintained by the University of Utah and the Veterans Health Administration and is freely available to entities such as health care organizations and EHR developers. LITE is an essential piece of OpenInfobutton, because it enables individuals with no information technology (IT) background to maintain the infobutton manager’s knowledge base. Although LITE is now being used to support infobutton implementation at several institutions, it is still very much a work in progress. To study users’ acceptance of LITE and to improve the usability of LITE, we conducted a multipart evaluation.

### Technical and Functional Background of LITE

LITE allows its users to define profiles of health care knowledge resource (such as MedlinePlus or UpToDate) and then define generic CSLs that describe the EHR contexts for which particular resources may be useful. A CSL is represented as a URL with a set of parameters specific to the resource. These parameters are defined in the HL7 Infobutton Standard and include information about the clinical settings (eg, inpatient unit or outpatient); EHR tasks (eg, medication order entry or problem list review); patient’s demographics (eg, gender, age, or language); user types (eg, physicians, nurses, or medical students); and a clinical concept of interest (eg, a medication, a problem, or a laboratory test result). Upon receiving a request from an EHR system, CSLs configured in LITE are used by OpenInfobutton’s infobutton manager to generate HL7-compliant URLs to various knowledge resources.

**Figure 1:**
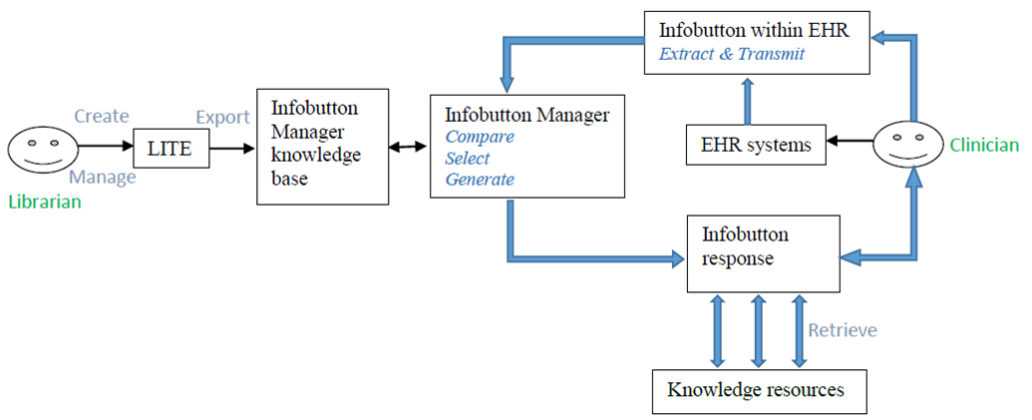
Diagram of Librarian Infobutton Tailoring Environment (LITE) and its clinical applications. EHR: electronic health record.

Figure 1 shows the relationships among LITE, the infobutton manager, EHR systems, LITE users, and EHR systems’ users. A simplified workflow of an infobutton includes the following steps: (1) an EHR user clicks on an infobutton within an EHR; (2) the infobutton evokes a Web browser that, in turn, evokes the parameters that were included by the EHR in the infobutton link and transmits the EHR context information to the infobutton manager; (3) the infobutton manager compares the EHR context with the CSLs in its knowledge base; (4) the infobutton manager selects CSLs that match the EHR context; (5) the infobutton manager instantiates the CSL with context-specific information (such as the concept of interest and patient demographics); and finally, (6) the infobutton manger passes a set of relevant and customized CSLs back to the Web browser in a standard XML format. LITE is used to build and manage a CSLs knowledge base in an easy-to-use and Web-based environment. LITE can be utilized by different institutions. The CSLs and the knowledge resource profiles can be exported from LITE to the infobutton manager for integration with various EHR systems, which increase CSLs’ portability.

LITE has the following main functions, which correspond to the steps a user must complete to provide CSLs for the infobutton manager:

1. *Define institutional profiles:* Each institution may have different knowledge resource subscriptions, different preferences, and different types of EHR systems.
2. *Define resource profiles:* To create profiles for external knowledge resources. This includes the resource-base URL, whether the resource is HL7 compliant or not, and the context parameters and standard terminologies that are supported by the resource.
3. *Create CSLs:* To identify the contexts (ie, clinical tasks and patient’s characteristics) in which the infobutton manager should select a particular resource.
4. *Test CSLs in a simulated EHR environment:* A functional test application allows users to set up HL7 infobutton requests, submit requests to the infobutton manager, and inspect the response.

**Figure 2:**
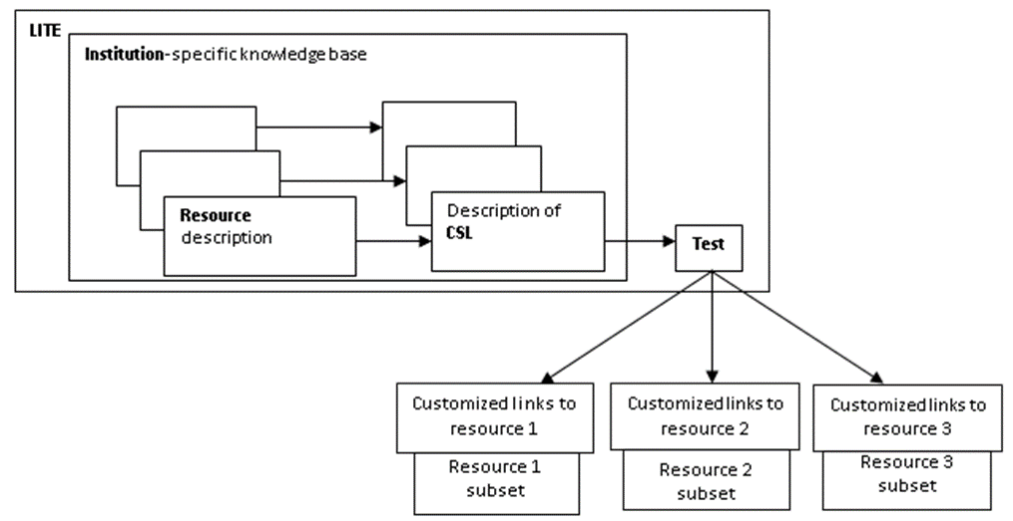
Librarian Infobutton Tailoring Environment (LITE) workflow diagram and the relationships among its main components. CSL: context-specific link.

**Figure 3:**
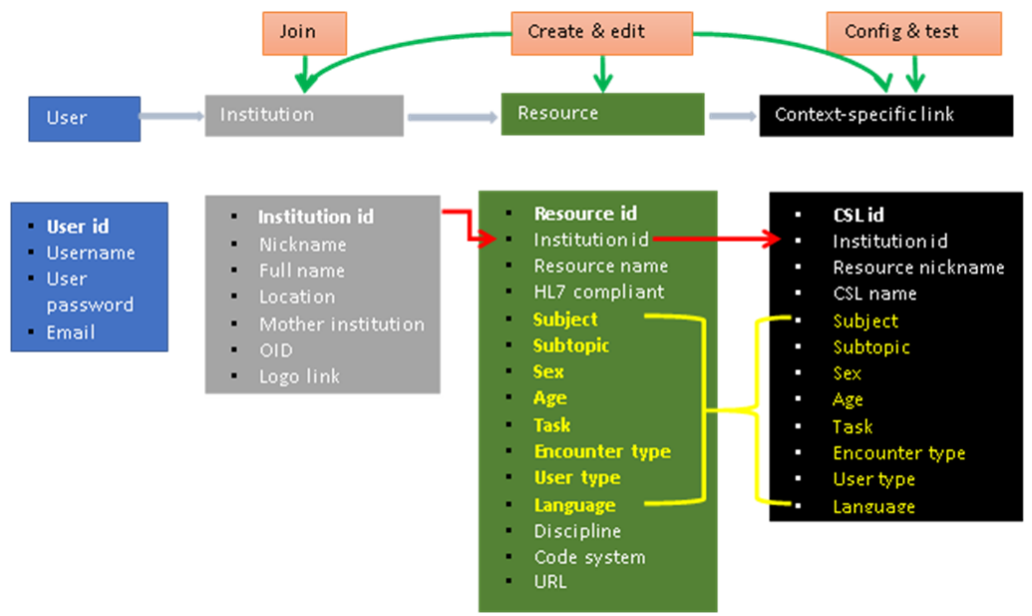
An overview of the main functionalities and structure of the Librarian Infobutton Tailoring Environment (the yellow highlighted fields are shared cross both resources and context-specific links [CSLs]).

Wizard-like applications guide the user through the creation of institutional profiles, resource profiles, and CSLs within LITE. Each step has a specific purpose, such as giving a name and a resource-base URL. An LITE user can review the content before saving the profile, and may modify the profiles after saving them. Figure 2 shows the main workflow within LITE and the relationships among the main components of LITE. Figure 3 shows the main functionalities and structure of LITE.

LITE is managed by Drupal, an open source content management platform. PHP and MySQL are used on the front end and back end, respectively, to support necessary programming functions.

To evaluate the usability and acceptance of LITE and more importantly to guide future development, we conducted a multiple parts evaluation and here we report how the evaluation was conducted and the related findings.

## Methods

The LITE evaluation has 2 main objectives: (1) to determine whether LITE fulfills its role as designed and (2) to identify ways to improve LITE’s usability. To achieve our objectives, we first surveyed users’ attitudes and usage experience about LITE. We then gathered direct and detailed feedback by observing actual usage of LITE on predetermined tasks, which corresponded to the 4 main components of LITE, and by interviewing users for their feedback about the ambiguous or less ideal parts. We also asked users their opinions about the current HL7-compliant parameters used in LITE for resource profiles and CSLs, which were selected by 3 field experts.

We used Kushniruk and Patel’s methodology [12] for the evaluation of clinical information systems. We also included follow-up questions to identify the specific reasons/problems that lead to negative comments in questionnaires and in observational study.

We conducted a pilot study before the formal evaluation: a semistructured interview and mock observational study (n=2). The pilot study helped identify LITE bugs, provided valuable information about the evaluation flow, and helped fine-tune the formal evaluation procedures.

**Figure 4:**
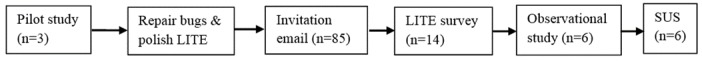
Librarian Infobutton Tailoring Environment (LITE) evaluation workflow (SUS: System Usability Scale survey; n refers to participants).

The formal LITE evaluation study was composed of 3 parts: a general survey, an observational study, and a System Usability Scale (SUS) survey. Figure 4 shows the study flow and includes the number of individuals who participated in each component of the study.

The LITE general survey focused on users’ experiences, their opinions about currently available functionalities, and their requirements and preferences for future development. In the observational study, we focused on the usability of the current version of LITE and identified specific functions with which users expressed dissatisfaction.

We invited all LITE users to participate in both parts of the evaluation. LITE users who completed the survey and expressed interest were recruited to participate in the observational study. Most LITE users are from the United States, but there are a few international users. Users include medical librarians, software engineers, hospital administrators, researchers, etc. All participants are recruited on a voluntary basis by 2 invitation emails without any incentives. Observational study participants received instructions regarding evaluation beforehand. The observational study was conducted via online sessions using WebEx, a Web conference tool. During the observational study, the researcher (XJ) reiterated the general purpose of LITE and the 4 designated tasks (define an institute, a resource, a CSL, test a CSL). Then the participant conducted the tasks one by one while sharing their work screen via WebEx. All the screen activities were recorded via BB FlashBack [13]; audio recording was optional. The researcher observed the whole process and was available to provide assistance during the study. The researcher also asked questions if the evaluator expressed hesitation, confusion, or interest at any point to identify the specific reason for confusion and capture every possibility that may improve LITE in future. A modified SUS survey followed the observational study. The main modification to SUS occurred if the evaluator had a negative answer; in this case, the SUS asked a follow-up question to specify the reason. The calculation of the SUS score was derived from the method described by John Brooke [14]. The general survey, SUS, and the evaluation instructions are attached as appendices to the manuscript. Both surveys were generated and managed within Survey Monkey.

For the video analysis, we used the following time-measurement criteria (using seconds as the measurement unit):

- *Start point:* The participant entered the first page to conduct a designated task;
- *End point:* The participant exited the last page for the designated task.

There were optional tasks (eg, modify an existing resource) in the evaluation, so not every participant conducted exactly the same number of tasks. The counts were based on tasks only, not on participants. For example, one participant might have created 2 or 3 CSLs, in which case each creation was counted independently. Therefore, final counts were task counts, not participant counts.

## Results

**Figure 5:**
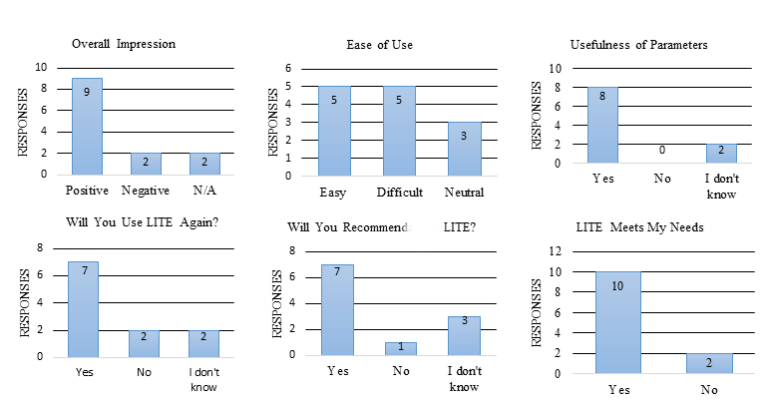
General Librarian Infobutton Tailoring Environment (LITE) evaluation survey results.

The response rate for the LITE general evaluation survey was 17% (14/85). The general evaluation survey results are summarized in Figure 5. Some of the original questions used numeric scales (1-10); however, for presentation purposes, we combined them into “yes/no” categories. For example, the original answers for “if LITE is easy to use?” and “if LITE meets your needs?” are a score from 1 (extreme negative) to 10 (extreme positive). We categorized the scores into yes (7-10), no (1-4), and neutral (5 and 6). The original answers about overall impression of LITE are free text, and we categorized them into yes (eg, it is wonderful, useful tool, looks great), no (eg, difficult to navigate, not clear to me...), and N/A (irrelevant answers).

For the observational study, all the designated tasks were conducted successfully except for one in which the participant did not successfully conduct a test of CSL due to an LITE server outage. Table 1 summarizes the time needed for completing each of the designated tasks. The average SUS score of LITE was 72.

**Table 1:** Time spent completing designated tasks in the observational study.^a^

Tasks		Min	Max	Average	SD	Median	Count
**Institution**							
	Create	0:58	8:06	2:58	2:22	2:27	7
	Change/verify	0:06	0:19	0:11	0:04	0:11	9
**Resource**							
	Create	2:20	13:48	5:35	4:41	3:57	5
	Modify	0:28	4:34	1:50	1:40	1:33	5
	Verify	0:05	4:05	1:02	1:19	0:34	8
**Context-specific link**							
	Create	2:05	8:37	4:27	2:47	3:08	6
	Modify/verify	0:27	2:46	1:31	1:10	1:18	3
Test of context-specific link		1:00	6:23	3:30	2:02	3:00	9

^a^Data are presented as minutes:seconds.

In Table 2, we summarize evaluators’ suggestions about LITE functionalities and interface presentation features from the general survey, observational study, and SUS survey. The frequent suggestions can be classified into the following categories: navigation, content layout and organization, functionalities, annotations and instructions, interface presentation features, and suggestions not specific to LITE. The main suggestions on improving LITE focused on consolidating information on one single page, sharing knowledge resources usage statistics information for decision making, providing more training and instructional materials, giving users more control of information display and organization, making required fields more evident in testing of CSLs module, providing additional documentation, and troubleshooting tips and giving more explanations about URL styles.

**Table 2:** Summarized suggestions from LITE evaluation studies.

Categories			Verbal suggestions/comments/feedback
**Navigation**			A little too much blending of information/hard to quickly pick my target
	**Content**		
		Organization	Available institutions (resources and CSLs) should be listed in the first page^a^
			It would be nice to have them all on 1 page in collapsible divisions so that you could jump between settings faster
		Adjustment	Performer discipline is probably unnecessary
			Race, ethnicity should be included
			Timestamps should be local to the user (or institution location)
	**Interface**		
		Visibility	Required fields need to be more evident in the test CSL module^a^
			Button fonts are too small
			Input boxes should be bigger with an obvious border
		Graphical design	To add visual connections between resources, CSL, and testing CSL
			“Continue” and “Previous” buttons should be switched
		Words usage preference	Prefer “Cancel” to “Quit”
	**Functionalities**		
		To make LITE more intelligent	Need more feedback in the testing component
			Be able to customize the views of resources and CSLs to show more fields from which data are being collected
			An editable table format as an alternative view for changing certain values or adding ones
		To improve LITE’s technical performance	Export resource/CSL profiles
			Can URLs be listed beside CSLs for troubleshooting purpose, with the search terms and other details?
Annotations			Subject (subtopic, URL style) needs a more comprehensive definition
Instructions			Progress bars for each wizard
			Additional documentation and troubleshooting tips^a^
Education and support			Can the webinar be recorded and make the webinar available online as a tutorial to help people learn how to use LITE?
			Need more help in defining resources and understand the technical features
Outside of LITE scope			Implementation of OpenInfobutton locally
			EHR integration forum, content, and videos

^a^The comments appeared repeatedly.

All general comments, such as “need an intuitive guidance,” that did not articulate the specific questions or problems were excluded from these tables. These are some positive comments by the evaluators: “LITE displayed all the information that was necessary in a clean interface”; “The ability to customize resources and create specific context situations around them is great”; “This greatly simplifies configuring resources for use with OpenInfobutton. It did a good job walking me through all the necessary setup steps”; “It’s straightforward and easy to use.”

## Discussion

### Interpretations of the Evaluation Results

The evaluators’ general impressions about LITE were quite positive. As a group, they thought that LITE allowed users to complete the main tasks successfully and quickly. The average time for observed users to complete each task was less than 6 minutes under a very relaxed test atmosphere. The time provides an objective measure for usage of LITE. Interview questions were common during the tests. The average SUS score of LITE was 72 (the average SUS score from literature is 68 [15]). According to Bangor et al [16] and Brooke [17], who examined more than 1000 SUS scores from different applications and technologies, the SUS 72 is in the good range (worst imaginable, awful, poor, OK, good, excellent, best imaginable). In our study, SUS is one measurement dimension we used to give readers a more comprehensive view about LITE and our main goal was to find evidence to guide future LITE development. Of the current infobutton standard parameters, users felt that age, sex, EHR task, subject, subtopic, encounter type (eg, inpatient, outpatient); user type (eg, provider, patient); and language were useful but that performer discipline was not necessary. Evaluators suggested that “race or ethnicity” and “geographic locations” should be added as new parameters. The latter has been included in the latest version of the HL7 Infobutton Standard and will be included in future versions of LITE.

LITE fulfills its primary role to build and manage CSLs for external resources in a user-friendly environment. The high rates of completion of designated tasks and positive feedback from evaluators confirm our confidence about LITE.

After we thoroughly read and analyzed the suggestions and comments, we classified them into the following categories: (1) users expected LITE to be more intelligent with a higher level of interaction, such as more feedback or troubleshooting tips from LITE during CSL tests; (2) users expected more control of the Web page display, such as the ability to customize the view of fields for resources and CSLs; (3) users desired data definitions of some data elements used in LITE to clarify the terminology; and (4) users desired education and support, such as troubleshooting tips.

### Significance of LITE

OpenInfobutton is one of the open-access resources that allows EHRs to comply with Infobutton standard to meet meaningful use requirements. Although meaningful use requirements are clearly defined, there still are gaps between implementation of certified EHR systems and meeting meaningful use requirements. One of them is how to populate and tailor infobutton manager’s knowledge base to optimize infobutton to suit the local health care settings. As part of the OpenInfobutton suite, LITE provides a mechanism for individuals at the EHR’s home institution to create and manage CSLs for OpenInfobutton’s infobutton manager. LITE is especially useful and helpful for clinical practices that lack sufficient IT support and professional health sciences librarians due to the graphical user interfaces. The centralized service, the graphical user interfaces, and wizard-like setup steps of LITE aim to save individual resources and efforts across different practices or institutions. Improving the user interface and usability of LITE will have a direct impact on helping practitioners and clinical settings adopting infobutton functionality within EHR systems to meet the meaningful use requirements.

### Significance of the Evaluation Study: Methods and Findings

We used multiple evaluation approaches (including 2 surveys, an interview, and an observational study) to gain a comprehensive view of LITE. In addition to multiple approaches, we gave evaluators opportunities to specify problems they encountered. Using this strategy, we obtained relatively high-quality input and feedback although we did not have a large sample size for the evaluation. Research shows that 5 participants can find at least 55% of the problems in usability tests and 10 participants can reveal over 80% of the problems [18] so further evaluation of LITE, when it has more users, is warranted.

Our analysis of the results revealed that users expect more interaction with LITE and more control of the interface display. A possible solution is to make LITE more intelligent, so that certain features, especially layouts and interfaces, are more readily adjustable according to users’ preferences. Because of the small sample size, the findings of the observational and usability sessions need to be considered with caution.

Although LITE does not contribute to evidence-based practice directly, LITE does play a critical role in configuring infobutton managers. Infobuttons are effective clinical decision support tool to help clinicians to conduct evidence-based practices. HL7 infobutton standard is required for EHR product certification and Infobuttons have been implemented in multiple sites [19,20]. A previous study [6] demonstrated that infobuttons can support clinicians in answering questions more efficiently, enhancing clinical decisions, and improving their knowledge with high positive impacts at the point of care. Therefore, LITE contributes to evidence-based practice in an indirect but necessary manner.

### Limitations of the Evaluation Study

One of the main limitations of this evaluation study is the low response rate. Two invitation emails are not sufficient to recruit evaluators. After the first invitation email, we should add incentives to attract more participants, which may help to recruit more participants. Because of the low response rate, we have a small sample size, and therefore, there are many limitations about how we can analyze the results and interpret them. For example, different age groups, different computer literacy levels, and different levels of LITE users (beginners and advanced users) may affect the evaluation results. We would be able to look at the factors that are associated with the evaluation results if we have a bigger sample size.

Another limitation can be introduced by the way we recruited evaluators: biased participants. Currently, all evaluators are recruited on a voluntary basis and the voluntary samples do not necessarily represent the real user population comprehensively. Well-stratified user groups and representations from each group may mitigate this type of bias; however, a larger sample size is a preliminary condition for such analysis.

How to measure the time and efforts saved using LITE cannot be answered by this evaluation study. To define a resource profile from scratch needs at least two tasks to be completed in LITE (to define an institute and to define a resource); and to define a CSL from scratch needs 3 tasks (to define an institute, a resource, and a CSL). This means that an LITE user would spend on average less than 18 minutes for each CSL configuration within LITE. Currently, we do not have a standard measurement of time that will be needed for an experienced knowledge expert to define a CSL usable by OpenInfobutton without LITE, such as to define XML file directly. Otherwise, the impacts of LITE can be claimed more specifically. For most nontech savvy users, dealing with graphical user interfaces may be far more pleasant than dealing with XML files directly. To find out how long it takes for an experienced knowledge expert to define a resource and a CSL can be an interesting separate study, which can be used as baseline data to evaluate the comprehensive impacts of LITE. Conservative estimation of LITE’s impacts should include the number of librarian-hours saved and not the level of work days saved.

Demographic data of the study participants are not available. We did not collect demographic data in surveys or in LITE registration. Now when we look back, computer literacy level may be a factor that is related to the evaluation results. A standard computer literacy test may thus be helpful before performing an SUS study.

### Challenges for Next-Stage Development

Prioritizing different suggestions and harmonizing controversial or contrasting opinions from different evaluators in a systematical manner create challenges as we consider ways to use this feedback in the next stage of LITE development. For example, having LITE break a task into detailed steps for the user to go through one by one is not as efficient for a tech-savvy user as completing all the steps within 1 page; however, other users complain that too much information increases the difficulty of navigation. Users’ computer literacy, their familiarity with LITE and background knowledge about LITE, infobutton managers, and EHR systems are all critical factors that may affect their views about LITE. However, how to deal with the challenges will be unavoidable for next-stage development. We may need a matrix to measure, prioritize, and calculate the different features and suggestions to guide the further development in a systematic manner.

The second challenge is finding a balance between fixed workflow and more alternatives (eg, to provide singular or multiple ways to access a specific page for initiating a task, to modify resources or CSLs). To increase its flexibility, LITE was designed to give users multiple ways to initiate creation and modification of resources and CSLs. However, this flexibility makes the workflow less simple, which may, in turn, confuse some LITE users who prefer a singular way for a task with few alternatives.

The third challenge is reaching a balance between trusting rational design decisions and relying on users’ feedback. When we designed LITE’s wizards for creating new CSLs or resources, every page (ie, step) has a “Continue” and a “Previous page” button, which let users navigate among different pages. For most LITE users, when they create a new CSL or a new resource, they have to click “Continue” to move forward. We placed “Continue” on the left of “Previous page” to make most of the clicks convenient. However, one evaluator pointed out that the 2 buttons should switch positions. Valid evidence about the optimal positions of the 2 buttons may need a large-scale observational study, which can detect the percentages of clicks for each button to complete 1 task precisely.

LITE is an important tool for furthering the adoption of infobuttons in EHR systems to meet meaningful use requirements. The evaluators were quite positive about LITE. LITE can be used to fulfill the original designated purposes successfully and quickly. The average time to complete a task is shorter than 6 minutes. It is an important challenge to meet all levels of users’ requirements within 1 output, so different layout plans for different users’ preferences (ie, more intelligent, information intense and more users’ control for advanced users and simpler, fixed workflow, and step-by-step wizards for beginners) may be a future direction for LITE.

## Abbreviations

CSL: context-specific link
EHR: electronic health record
LITE: Librarian Infobutton Tailoring Environment
SUS: System Usability Scale
